# Tax and noncommunicable diseases attributable to tobacco and alcohol consumption in 5 Sub-Saharan African countries

**DOI:** 10.3389/fpubh.2024.1464897

**Published:** 2025-01-10

**Authors:** Machiru Moyo, Gowokani Chijere Chirwa, Thomas Nyirenda, Beatrice Lydia Matanje

**Affiliations:** ^1^Economics Department, University of Malawi, Zomba, Malawi; ^2^North-West University, Department of Economics, Potchefstroom, South Africa; ^3^European and Developing Countries Clinical Trials Partnership, Medical Research Council, Cape Town, South Africa; ^4^Clinical Research Education and Management Services (CREAMS), Lilongwe, Malawi; ^5^Global Health Department, Epidemiology and Statistics Unit, Stellenbosch University, Stellenbosch, South Africa; ^6^National AIDS Commission, Executive Management Division, Lilongwe, Malawi

**Keywords:** fiscal policy, non-communicable diseases, WHO best buys, health promotion, tobacco taxation, alcohol taxation, Malawi

## Abstract

**Background:**

Increased taxation on alcohol and tobacco is among the cost-effective measures used to deal with the burden of noncommunicable diseases (NCDs) globally. Despite adopting such efforts, the impacts of taxation on alcohol and tobacco are yet to be fully understood.

**Objective:**

The study's objective is to find empirical evidence regarding changes in the NCD mortality rate associated with changes in the tax rates of tobacco and alcohol.

**Methods:**

The study adopted the System Generalized Method of Moments (SGMM) to explore the relationship between levels of taxes and NCD mortality rates. The SGMM allowed the inclusion of the dependent variable as an explanatory variable, assuming reverse causality was assumed.

**Results:**

There appears to be a negative relationship between increased taxes and the rates of NCDs. Specifically, we provide empirical evidence supporting the negative association between taxes on alcohol and tobacco cigarettes and the mortality rates from NCDs, which aligns with the propositions advocated by the World Health Organization (WHO). Additionally, the interaction between alcohol taxes on spirits and beer indicates a possibility of complementarity, consistent with taxation principles. Notably, we also observed that higher tobacco cigarette prices are negatively associated with NCD mortality rates.

**Conclusion:**

The results indicate that increasing taxes on major health risk factors is necessary to reduce non-communicable diseases (NCDs). Implementing these tax increases will likely help achieve Sustainable Development Goal 3.4, which aims to reduce NCD mortality by one-third by the year 2030.

## Introduction

Classical production theory postulates that labor and capital are vital to production (1). Of much significance is the fact that labor uses capital for production processes. Noncommunicable diseases (NCDs) may affect production by reducing labor productivity (2, 3). NCDs have negative health implications and are significant contributors to reduced economic progress and productivity. NCDs result in long-term health issues as they lead to reduced quality of life, require long-term treatment and care among sufferers, introduce lifestyle changes, or, in some extreme conditions, necessitate palliative care, which also has negative implications on careers. This is a threat to economic progress as human productivity is negatively affected due to lost income that consequently temporarily or permanently cripples the labor force of the economy (4).

Global statistics show that NCDs are the leading cause of death and disability worldwide (5). NCDs are responsible for 74% of global deaths, claiming the lives of 15 million people prematurely between the ages of 30 and 70 (5). Since each death results in a loss of income, NCDs have a significant economic cost (3, 6). Without effective interventions, the World Health Organization (WHO) predicts that the number of NCD-related deaths will sharply increase to 55 million by 2030 (5).

Regarding their effect on productivity, NCDs have surpassed infectious diseases in the African Region, making up about 37% of the total disease burden (5). The Sub-Saharan region experiences an epidemiological change in the disease burden, with a sharp 67% increase between 1990 and 2017 (37). Unlike communicable diseases that arise due to a germ (viral, bacterial, or fungal) infection, few NCDs are genetically inherited, while most of them occur due to lifestyle or environmental factors (7). Among lifestyle factors, long-term use of tobacco and alcohol is identified as one of the main risk factors that are associated with the burden of NCDs (5).

The WHO has implemented a comprehensive Global Action Plan for the Prevention and Control of NCDs that runs from 2013 to 2030, aiming to ease the burden of NCDs. Specifically, the third objective of this plan focuses on addressing risk factors and underlying social determinants. To achieve this, the plan emphasizes the reduction of tobacco and alcohol use, as they both significantly contribute to the prevalence of NCDs. The proposed plan aims to contribute to achieving global targets of a 30% reduction in tobacco use and a 10% reduction in harmful alcohol consumption. One of the effective measures to reduce the demand for these products is increased tax imposition to reduce affordability (38).

Global statistics indicate that more than half of the 3 million annual deaths attributable to alcohol use are from NCDs, including cancer. Tobacco accounts for over 8 million deaths every year. More than 7 million of those deaths are the result of direct tobacco use, while around 1.2 million are the result of non-smokers being exposed to passive or second-hand smoke (5). The principle of heavily taxing tobacco products and alcohol is to reduce consumption, thereby lowering these risk factors that bring about NCDs (5). The issue of taxing tobacco goes way back to when classical economist Adam Smith in his book “*The Wealth of all Nations”*, posits that “*Sugar, rum, and tobacco, are commodities which are nowhere necessaries of life, which have become objects of almost universal consumption, and which are, therefore, extremely proper subjects of taxation,”* (8). The rationale for taxing tobacco in these primary days was to raise government revenue. The literature identifies other proposed reasons for taxing tobacco, one of which, among the many, is to promote public health by reducing morbidity and mortality effects associated with smoking. This exciting context reflects how the debate on tobacco taxation has evolved (9).

The WHO implemented the Framework Convention on Tobacco Control (FCTC) as a commitment to deal with the global tobacco epidemic and prioritize public health and wellbeing (5). MPOWER stands for; Monitor tobacco use and Prevention policies; Protect people from tobacco smoke; Offer help to quit tobacco use; Warn about the dangers of tobacco; Enforce bans on tobacco advertising, promotion, and sponsorship and; Raise taxes on tobacco, has been introduced as a technical package designed to help countries implement demand-reduction measures for tobacco.

Now, the nexus between NCDs and taxation is dynamic. According to the WHO Steps Surveillance (5), tobacco use imposes a risk of developing cancer, especially lung cancer, heart disease, stroke, and diabetes. It becomes a threat to passive smokers, who are between 20% and 30% at risk of developing lung cancer and other chronic respiratory diseases. A recent study conducted on the “Global Burden of Cancer” reports that alcohol consumption accounts for 4% of specific cancers (40). Since these products are sold in a market governed by the forces of demand and supply, tampering with their prices through taxation reduces their purchase. This implies that the consumption of these products will be lowered, as will the risk of developing NCDs like lung cancer, diabetes, hypertension, and chronic respiratory diseases. Furthermore, lowered risk will be reflected in lower NCD mortality rates (10–13).

There has been an interest in this research nexus in the past. Most of it has been from not within the African region but the Western countries, with most of them concluding that the taxes are effective in reducing NCD mortality rates (6, 39–43). Mustapha et al. (14) researched the effects of increased tobacco taxation and pricing on smoking prevalence in Africa. The authors found that increased taxation and pricing have significant negative effects on the prevalence of smoking. The former had a negative effect on the prevalence of smoking by 0.25% to 0.36%, while the latter had a negative effect on the prevalence of smoking by 0.11–0.14% (14, 15). Similarly, extensively researched the tax structure and prices of cigarette excise. The study demonstrates that more superficial tax structures would be much more effective in reducing cigarette smoking than tax structures that are more complicated, as there is a greater likelihood to switch to cheaper brands whenever a tax increase is imposed. In addition to the aforementioned, it was found that alcohol taxation consistently reduces consumption. Alcohol prices and taxes are inversely related to drinking (16).

Alcohol control strategies mirror those of tobacco control; however, there is little empirical evidence on the possible effects of increased alcohol taxation on preventing NCD deaths. The present study builds from gaps in the literature above to find empirical evidence regarding changes in the NCD mortality rate associated with changes in the tax rates of tobacco and alcohol. From this primary objective, we specifically sought; to assess the association between tobacco tax and NCDs mortality rates; assess the association between alcohol tax and NCDs and lastly; investigate the persistence of past NCDs mortality rates. We used data from five Sub-Saharan African countries to determine whether increased or heavy taxation breathes new hope into these countries' efforts to combat NCDs as we approach the Sustainable Development Goal (SDG) 2025 target of a 25% global reduction in NCD mortality rates.

Undertaking this study is important. With the current statistics on NCDs, there is a high possibility that the target to reduce NCD mortality by one-third by 2030 might not be achieved (SDG 3.4 target). Deaths related to NCDs affect SDG8 of economic growth, in particular target 8.2, because of the long-term health effects that impede productivity. This has implications for the poverty levels in the economy and SDG1 of no poverty target 5. A failure to reduce poverty will likely bring inequalities among communities as per SDG10.2.

## Methods

We used the System Generalized Methods of Moments (SGMM) to estimate and assess the relationship between changes in the death rate from NCDs in five Sub-Saharan countries relative to the changes in tax rates on alcohol and tobacco products, tobacco cigarette prices, and screening rates. Urban growth and income per capita have been used as instruments to control for potential endogeneity and the validity of the estimates. Furthermore, SGMM allows for the dependent variable to be included as an explanatory variable if there are any suspicions of reverse causality. Furthermore, GMM does not rely on assumptions about the normality of the data distribution. It can be applied to a wide range of linear and nonlinear models without being limited by specific distributional assumptions (17–20). We specify the following relationship below:


(1) $$\begin{array}{l} NCDM_{it}=f\left(Tax_{it},K_{it},\varepsilon_{it}\right) \end{array}$$

(2) $$\begin{array}{l} NCDM_{it}={{\alpha_{0}+\beta_{1}NCDM_{it-1}+\beta }_{2}Tax}_{it}+{\beta_{3}K}_{it}+\varepsilon_{it} \end{array}$$

Where *K*_*it*_ is the vector of the controlled variable, and α_0_ is the intercept.

*NCDM*_*it*_ is the mortality rate of noncommunicable diseases and is expressed in percentages.

*Tax*_*it*_ is the excise tax on the most sold brands of tobacco cigarettes, and alcohol specifically beer and spirits.

*K*_*it*_ is the vector of control variables including; tobacco cigarette prices, screening rates, urban growth rate, and income per capita.

β_1_−β_3_ are coefficients of explanatory variables.

ε_*it*_ is the error term.

### Data

The data used in this study was sourced from the World Health Organization Database, World Bank Database, and WHO Health Observatory. The data spans from 2008 to 2022 and includes information on NCD mortality rate, income per capita, urban growth rate, and health taxes. The NCD mortality rate, income per capita, and urban growth rate for each of the five nations are based on data from the World Bank, while the health tax information is extracted from the WHO Database. The WHO Health Observatory Data Base (health taxes) contains information on alcohol and tobacco cigarette taxes and prices. The tax used in this study is the excise tax from the most sold brand of these products. While alcohol taxes only cover 2022, tobacco cigarette taxes and prices go from 2008, 2010, 2012, 2014, 2016, 2018, 2020, and 2022.

For analysis, we assumed that the tobacco cigarette tax data and prices remained in effect for the other years for which there was no data, assuming they haven't been changed. Alcohol taxes have not been proposed because the data only covers a single period. A dummy variable representing screening rates was established to supplement the independent variables. The dummy variable is categorical, with values of 1 for lower screening rates, 2 for medium screening rates, and 3 for high screening rates.

## Results

**Figure 1:**
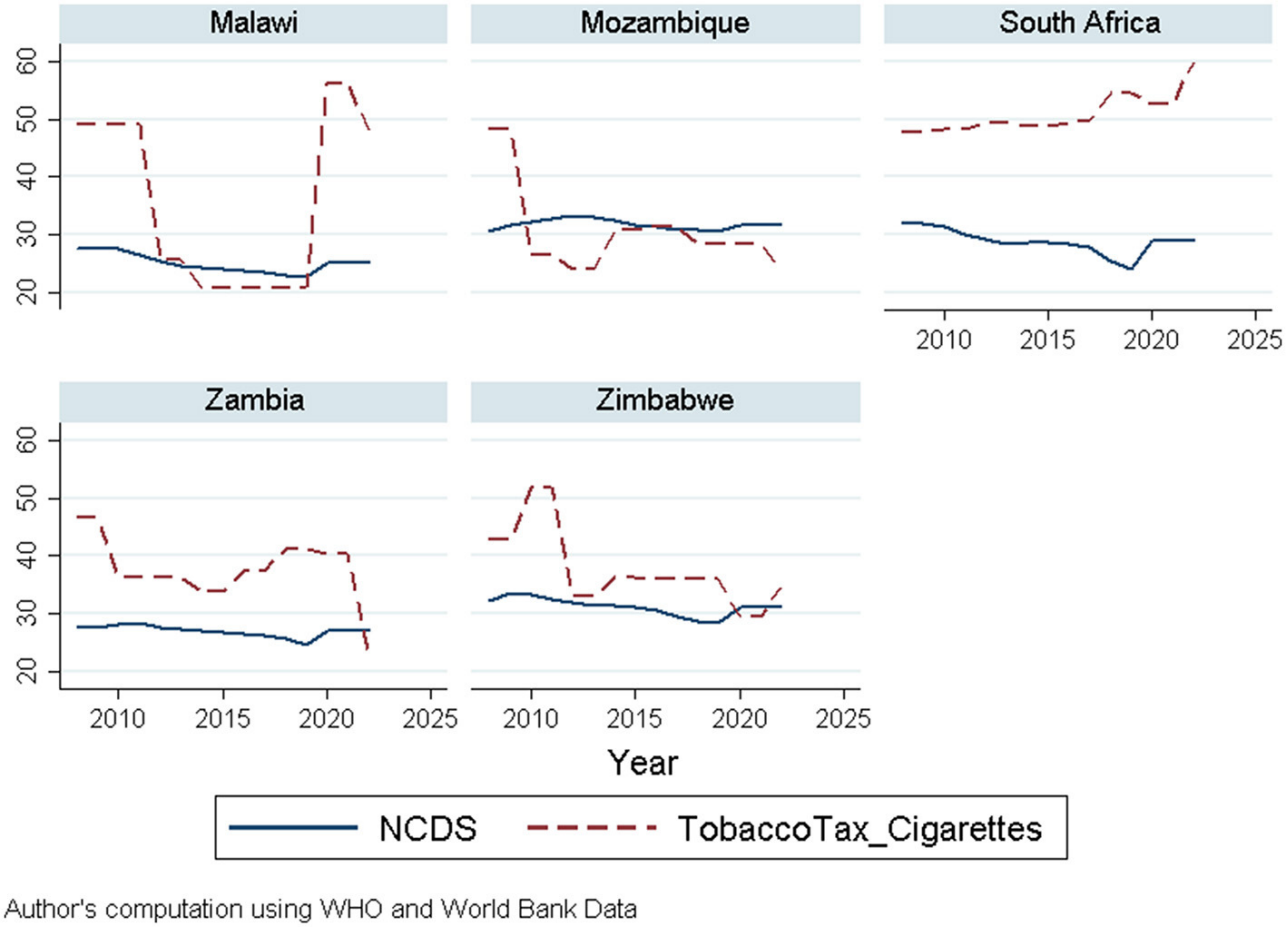
NCDs mortality rates and tax on tobacco cigarettes by country for the years 2008–2022.

The visual presentation of NCD mortality and taxes on tobacco cigarettes within the implementation period of the tax (2008–2022) in Figure 1 below shows a rise and fall in NCD mortality and tax rates on tobacco cigarettes over the years. Malawi and Mozambique exhibit the same pattern; however, Malawi's graph indicates a drastic increase in tobacco cigarette tax around 2020. Zambia and Zimbabwe show fluctuations in both NCDs and tax rates, while South Africa depicts a stable tax rate but rising NCDs, suggesting consistency in tax policy implementation.

As much as these countries are members of the WHO Framework Convention on Tobacco Control (FCTC) which aims to fight the tobacco pandemic, the fluctuations in the graph of tax on tobacco cigarettes may not solely be attributed to the desire to reduce the burden of NCDs mortality rates. Policy reforms are among the various factors that alter tax changes. The policy reforms that might have influenced cigarette taxation during the 2008 to 2022 period are summarized in Table 1.

**Table 1:** Tax reforms by country within the period of 2008–2022.

**Country**	**Reform**
Malawi	▪ In 2008, the Malawi government introduced low taxes on cigarettes containing 70% of Malawi tobacco, tax stamps, and a change from ad valorem to a specific tax. The rationale was to curb smuggling and develop a strong tobacco sector in Malawi. ▪ In 2022 the added tax on cigarettes was suspended.
Mozambique	▪ In 2009, the excise tax was adopted. It introduced specific tax rates and ad valorem for cigarettes. ▪ In 2010, the tiered specific excises for cigarettes and mixed (ad valorem and specific) excises for other tobacco were put in place. ▪ In 2013-2015, there was an increase in excise tax on cigarettes for public health concerns and revenue generation.
South Africa	▪ During the 2008 period, there was a 52% increase in the total excise tax aimed to control tobacco. ▪ In 2020-2021, there was an 8% increase in excise duty. The rationale was to grow government revenue after the COVID-19 crisis.
Zambia	▪ In 2020, the tax on tobacco cigarettes declined because of the tiered tax structure which gives a preferential excise tax rate for domestically manufactured cigarettes.
Zimbabwe	▪ In 2020, the Zimbabwe government increased excise duty on cigarettes to help the health sector.

### Descriptive statistics

Descriptive statistics reflect the distribution and diversity of variables. The statistics give insight into the average value, variability, and range of each variable. Table 2 displays descriptive statistics for all variables.

**Table 2:** Summary statistics.

**Variable**	**Obs**	**Mean**	**Std. Dev**.	**Min**	**Max**
NCDS mortality	75	28.748	2.951	22.6	33.5
Tobacco tax cigarettes	75	38.476	10.859	20.68	60.09
Alcohol tax beer	75	2.141	8.451	0	40.64
Alcohol tax spirits	75	3.247	12.354	0	59.55
Income per capita	75	1,614.437	1,999.285	19.129	7,006.261
Tobacco cigarettes prices	75	3.46	1.788	0.96	7.04
Urban growth rate	75	3.259	1.306	0.254	4.856

The variation in NCD mortality rates across the five countries is minimal. There is higher variability in cigarette tax rates with a standard deviation of 10.859, indicating a higher dispersion around the mean. The minimum and maximum values show a wide range of tax rates for cigarettes with a minimum of 20.68 and a maximum of 60.9. Alcohol tax on beer shows a considerable variation in beer taxes. The standard deviation of alcohol tax on spirits suggests significant variation across countries. There are wide disparities in income per capita across countries. However, there is moderate variability in tobacco cigarette prices and urban growth rate.

### Econometric findings

After conducting all the necessary diagnostic tests, the analysis investigated the changes in NCD mortality rate relative to the changes in tax rates of alcohol and tobacco products using the system SGMM estimator. The lagged dependent variable has been included as a regressor to account for any persistence correlation. Urban growth and income per capita have been used as instruments and are certified valid by the Sargan Test. They help to account for endogeneity and improve the validity of estimates. The results in Table 3 display the SGMM output.

**Table 3:** System GMM output.

**NCDS mortality**	**Coef**.	**St. Err**.	**t-value**	***p*-value**	**95% Conf**	**Interval**	**Sig**
Tobacco tax on cigarettes	−0.042	0.007	−6.17	0.000	−0.055	−0.028	^***^
Alcohol tax on beer	0.115	0.048	2.40	0.016	0.021	0.209	^**^
Alcohol tax on spirits	−0.012	0.004	−2.64	0.008	−0.02	−0.003	^***^
Tobacco cigarettes prices	−0.887	0.051	−17.38	0.000	−0.0987	−0.787	^***^
Interaction of tobacco tax om cigarettes and tobacco cigarettes prices	0.018	0.002	9.63	0.000	0.014	0.021	^***^
Medium screening rates	−0.461	0.219	−2.10	0.036	−0.890	−0.031	^**^
High screening rates	0.164	0.065	2.53	0.011	0.037	0.292	^**^
Interaction of alcohol tax on beer and spirits	−0.002	0.001	−2.20	0.028	−0.0037	−0.0002	^**^
Lagged NCDS	0.766	0.028	27.85	0.000	0.712	0.820	^***^
Constant	8.995	0.928	9.69	0.000	7.175	10.814	^***^
Mean dependent var	28.651		SD dependent var			2.983	
Number of obs	70		Chi-square			434.186	

^***^p < 0.01 (highly significant), ^**^p < 0.05 (significant), ^*^p < 0.1 (marginally significant).

A one percent increase in tobacco-cigarette tax is associated with a decrease in NCD mortality rates by 0.0416%, holding all other factors constant. The coefficient is statistically significant at 5%. This conforms to public health goals, as higher cigarette prices can discourage smoking and reduce health risks that are attributed to NCDs. The negative results may also reflect the issues of elasticity as WHO reports indicate that the elasticity of tobacco cigarettes ranges from 5 to 8% in low- and middle-income countries, while in high-income countries, it is about 4%. A positive coefficient of alcohol tax on beer implies that a one percent increase in excise tax on beer is associated with an increase in NCD mortality by 0.1152%, holding all other factors constant.

The coefficient is statistically significant under the 5% level. This finding is intriguing and might warrant further investigation, as it could derive from other factors related to beer consumption. A negative coefficient of alcohol tax on spirits suggests that a one percent increase in excise tax on spirits is associated with lower NCDS mortality rates by 0.0116%, all things being equal. The coefficient is statistically significant at the 5% level. The negative result may also imply that spirits tend to have higher alcohol content, so this result might reflect differences in alcohol consumption patterns. A negative coefficient of tobacco cigarette prices indicates that higher prices are associated with lower NCD mortality rates by 0.08868%. The coefficient is statistically significant.

The joint effect of alcohol tax on beer and spirits is associated with a decrease in NCD mortality rates by 0.002%. The coefficient is significant at a 5% level. The negative association might also be attributed to whether these products are complements. The combined effect of increasing both cigarette tax and prices is associated with an increase in the NCD mortality rate by 0.018%. The coefficient is significant at the 5% level.Medium screening rates are negatively associated with NCD mortality rates. Higher screening rates are positively associated with NCD mortality rates as opposed to lower and medium screening rates. The result is expected mainly in disease epidemiology; when screening tools are introduced, or the old ones are improved, there is an increment in the death rate of the disease. This increase is due to the introduction of new tools. This suggests that in the past, the diseases were present in their respective countries, but no one picked them up. Both screening rates for dummies are significant at a 5% level. Past NCDs have a positive association with current NCDs by 0.7664%. This suggests a prevalence in NCD mortality rates. They are significant at the 5% level. The constant value of 8.9948 represents the expected NCD mortality rates when all other variables are zero. It is statistically significant.

## Discussions

This study empirically investigated the relationship between NCD mortality rates relative to changes in tax rates on tobacco cigarettes and alcohol (beer and spirit) while controlling for tobacco cigarette prices, screening rates, income per capita, and urban growth rates. This was done by collecting data for five sampled Sub-Saharan countries from 2008 to 2022. The tax data used is about the excise tax on the most sold brands extracted from the World Health Organization. We find that increased taxes on tobacco or alcohol or both seem to have a negative significant association with NCDs.

The empirical evidence of the negative association of alcohol and tobacco cigarette taxes on the mortality rates of NCDs confirmed the propositions that have been advocated by the WHO (36). Also, the use of the fiscus in dealing with the NCDS scourge has been reported in several studies to be effective and a negative relationship has also been found (21, 22). Furthermore, the negative association was reported in the Caribbean (23), Nepal (24), Peru (25), and many developed countries such as those in Europe (42).

The interaction of alcohol tax on spirits and beer tax conforms to the principles of taxation and reflects a possibility of complementarity. On the other hand, the interaction of tobacco tax on cigarettes and tobacco cigarette prices is counterintuitive. One interesting finding of these results is the significance of the intercept, indicating the presence of additional factors that may have a significant association with NCD mortality.

We also noticed that increased tobacco cigarette prices have a negative association with NCD mortality rate. This finding also agrees with the finding of Pearl et al. (12), who conducted a study on the effects of tobacco taxation and pricing on smoking behavior in high-risk populations. The effect may probably come from the substitution effect as people may switch to consuming healthy goods. This may be given that the household budget may be effectively reduced in relative terms due to the price increase (26). This supports rational choice theories, in which people weigh the benefits of a decision and opt for the option that yields the most beneficial outcome that maximizes utility (27).

Related to NCD screening, the categorical screening rates dummy variables imply that high screening rates unravel some NCD mortality rates that have not been picked up due to potential underdiagnosis. The importance of NCD screening in similar scenarios was reported among healthcare workers in South Africa (28) and for hypertension in Malawi (29). The screening may be an essential preventive strategy in areas like the countries analyzed. This comes about because these countries' have low capacity to adopt a curative approach due to a lack of adequate facilities and low budget (30), except for South Africa, which may finance a large part of its health budget (31). Furthermore, the study showed that using lagged NCD mortality rates as an explanatory variable hints that previous NCD mortality rates may influence present or future NCDs. This is likely to be influenced by policy interventions that would impact the outcome of NCDs' present or future mortality rates.

Having said the above, it is imperative to indicate that the success of the tax intervention depends on taking a holistic approach. Such is the case because people trade-off, and there is a temporary or permanent coping mechanism as humans are rationing and responding to price incentives, a phenomenon that was already observed in Malawi, where alcohol consumption overcrowded the consumption of other household commodities (32). Consequently, the alcohol or smoke addicts reduce the consumption of other necessities (44). To model the demand for these products, addiction-related issues must be taken into consideration because people with addiction would rather modify their spending in other areas to make up for the decrease in their present consumption than alter their behavior in the face of higher taxation. Further, different stakeholder perceptions may matter as they may jeopardize the policy process- a finding established in Nepal (22).

Since there is no one-size-fits-all solution, differential taxes that target particular populations may be considered. In nations such as South Africa, where a varied range of racial backgrounds predominate, it is customary for individuals to smoke and drink alcohol (33–35). Effective implementation of tax interventions requires consideration of all these factors. In addition, since these levies provide the government with income, there should be consideration that the tax revenue generated should be channeled toward funding health initiatives related to NCDs and the treatment of current NCDs.

There are possible solutions for improving the effectiveness of these interventions, but there are also potential blind spots. Alcohol and tobacco products are taxed in the formal sector but not in the unregulated informal sector, where government supervision is minimal. This poses a challenge in some countries analyzed here, which comprise a large informal sector. Furthermore, WHO proposes taxes on the most sold brands of tobacco products and alcohol. An intervention that might compel people to switch to local brands if a tax hike makes these products costly. It is important to bear in mind that the goal of tax interventions is to ensure that the consumption of these goods is reduced to the point where there are no viable substitutes while balancing the impact on household income for countries like Malawi where these products, specifically tobacco has economic significance, without compromising public health goals. In addition, differences in tax distribution across countries might also yield significant differences in the reduction of modifiable risk factors.

## Conclusion

Our interest was to assess the effect of tax and noncommunicable diseases attributable to tobacco and alcohol consumption in five Sub-Saharan African Countries. On our first objective, we successfully established a negative significant association between tobacco tax and noncommunicable diseases (NCDs), indicating that increased tobacco taxation is linked to the reduction of NCDs mortality rates. Regarding the second objective,our analysis revealed a substantial negative association between alcohol tax and NCDs, demonstrating that higher alcohol taxation is associated with decreased NCDs. As for the last objective -to investigate the persistence of NCDs, we incorporated NCDs as a regressor in the model. The results show a positive significant association between past and current NCDs, thereby confirming the persistence of NCDs.

The results of this study call for more research to address some problematic areas raised, such as variations in demand responsiveness to price changes. In addition, the intercept in the regression analysis of this study shows a strong association with the dependent variable. The research should assist policymakers in investigating other factors influencing NCDs. This also includes the reaction of the tobacco and alcohol industries to increased taxation. This will be extremely important when creating interventions.

## Data Availability

The datasets presented in this study can be found in online repositories. The names of the repository/repositories and accession number(s) can be found in the article/Supplementary material.
